# β1-Integrin plays a major role in resveratrol-mediated anti-invasion effects in the CRC microenvironment

**DOI:** 10.3389/fphar.2022.978625

**Published:** 2022-09-02

**Authors:** Aranka Brockmueller, Anna-Lena Mueller, Parviz Shayan, Mehdi Shakibaei

**Affiliations:** ^1^ Musculoskeletal Research Group and Tumor Biology, Faculty of Medicine, Institute of Anatomy, Chair of Vegetative Anatomy, Ludwig-Maximilians-University Munich, Munich, Germany; ^2^ Department of Parasitology, Faculty of Veterinary Medicine, University of Tehran, Tehran, Iran

**Keywords:** β1-integrin, CRC, inflammation, invasion, metastasis, NF-κB, tumor microenvironment, resveratrol

## Abstract

**Background:** Tumor microenvironment (TME) is one of the most important factors in tumor aggressiveness, with an active exchange between tumor and other TME-associated cells that promotes metastasis. The tumor-inhibitory effect of resveratrol on colorectal cancer (CRC) cells has been frequently reported. However, whether resveratrol can specifically suppress TME-induced CRC invasion *via* β1-integrin receptors has not been fully elucidated yet.

**Methods:** Two CRC cell lines (HCT116, RKO) were cultured in multicellular, pro-inflammatory 3D-alginate TME cultures (containing fibroblasts, T-lymphocytes) to investigate the role of β1-integrin receptors in the anti-invasive and anti-metastatic effect of resveratrol by antisense oligonucleotides (ASO).

**Results:** Our results show that resveratrol dose-dependently suppressed the migration-promoting adhesion adapter protein paxillin and simultaneously enhanced the expression of E-cadherin associated with the phenotype change of CRC cells, and their invasion. Moreover, resveratrol blocked TME-induced phosphorylation and nuclear translocation of p65-NF-κB, which was associated with changes in the expression pattern of epithelial-mesenchymal-transition-related biomarkers (slug, vimentin, E-cadherin), metastasis-related factors (CXCR4, MMP-9, FAK), and apoptosis (caspase-3). Finally, transient transfection of β1-integrin, in contrast to knockdown of NF-κB, abrogated most anti-invasive, anti-metastatic effects as well as downstream signaling of resveratrol, resulting in a concomitant increase in CRC cell invasion, indicating a central role of β1-integrin receptors in the anti-invasive function of resveratrol.

**Conclusion:** These results demonstrate for the first time that silencing β1-integrins may suppress, at least in part the inhibitory effects of resveratrol on invasion and migration of CRC cells, underscoring the crucial homeostatic role of β1-integrin receptors.

## Graphical Abstract

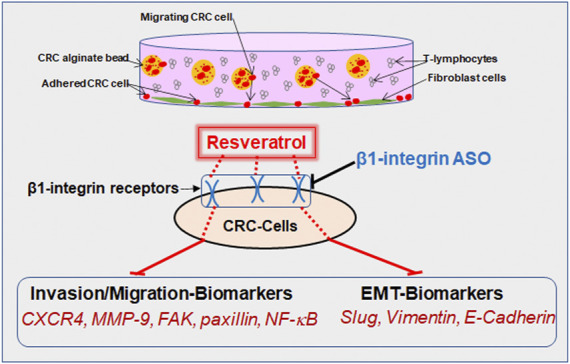

## Introduction

Cancer is one of the most commonly diagnosed diseases that causes many deaths among populations worldwide. According to global cancer statistics, there were over 19 million new cancer cases and 10 million cancer deaths globally in 2020 (Sung et al., 2021). Herein, colorectal cancer (CRC) represented 10% and the total number is expected to further increase within the next 20 years (Sung et al., 2021). In fact, CRC is the second leading cause of cancer deaths in the United States (Siegel et al., 2020). It is known that not only patients of advanced age are at risk, but also unhealthy lifestyle, sedentary behavior, excessive obesity or smoking play a decisive role in the development of cancer disease (Siegel et al., 2020). Moreover, despite the rising prevalence of screening and the growing number of colonoscopies (Siegel et al., 2020), more investment is needed to detect CRC at an early stage. Indeed, it has been reported that when a CRC is first diagnosed, up to 25% of patients already have been developing metastases, and as the disease progresses, as many as half of all affected individuals develop metastases (Vatandoust et al., 2015).

In that regard, metastatic tumors are cells that have spread outside of the colorectal tract *via* the blood or lymphatic system and are then located predominantly in the liver or lungs. The metastatic process, which is one of the six hallmarks of cancer, is usually a sign of a high-progressive disease and explains why patients die more often from secondary than from primary tumors (Koklesova et al., 2020). Furthermore, these lethal complications can also be triggered by metastases damaging affected organs, leading to dysfunction, organ failure, and ultimately to death in patients.

As inflammatory processes are one of the major underlying mechanisms of cancer diseases, colorectal tumors also contain large numbers of cells that can trigger excessive immune responses and cytokine production, leading to increased activation of pro-inflammatory and oncogenic transcription factors such as nuclear factor kappa-light-chain-enhancer of activated B-cells (NF-κB) (Rasool et al., 2021). In the advanced cascade of tumor invasion and metastasis, epithelial–mesenchymal transition (EMT) plays an essential role, which describes the process of cells changing their polarity from epithelial to mesenchymal characteristics, thus are able to migrate. While this transformation is a physiological process during the embryonic phase, it is considered highly pathological in the context of cancer development and migration (Koklesova et al., 2020). In addition, there are numerous signs of EMT, such as reduced E-cadherin expression acting as an epithelial marker with simultaneously increased vimentin expression as a mesenchymal marker (Koklesova et al., 2020). Moreover, it has been reported that the inflamed multicellular microenvironment of CRC significantly promotes expression of EMT proteins and transcription factors such as slug (Buhrmann et al., 2015). Indeed, this complex disease process requires therapy that attacks various levels in a multi-faceted manner, thus it is advisable to incorporate the power of nature-derived substances into treatment, such as the polyphenol and phytoalexin resveratrol. Resveratrol was found as a stilbenoid in several plants such as grapevines and peanuts, where it is synthesized in order to protect plants against pathogens (Fritzemeier et al., 1983; Hain et al., 1993).

Based on preclinical studies in CRC, resveratrol has already been shown promising effects in the subject of anti-inflammatory, anti-proliferative and anti-invasive properties in tumor cells (Brockmueller et al., 2022), and its clinical application is currently under intense investigation (Brockmueller et al., 2021). Thus, previous studies by our group among others have shown that resveratrol is able to suppress the dangerous and tumor-promoting cross-talk between CRC cells and stromal cells in a pro-inflammatory tumor microenvironment (TME) *in vitro*. Furthermore, resveratrol, as a multi-target molecule has been reported to interfere and negatively affect NF-κB signaling pathways, which is one of the major inflammatory transcription factors and markers promoted by the TME, that was shown to affect invasion behavior matrix metallopeptidase 9 (MMP-9) and metastasis process chemokine receptor type 4 (CXCR4) in CRC cells (Bergman et al., 2013; Buhrmann et al., 2016; Suh et al., 2018; Buhrmann et al., 2020).

Moreover, the prevention of EMT mechanisms in tumor cells is one of the most important goals in cancer research and therapy and has been increasingly attributed to be of fundamental importance. In this context, resveratrol has been frequently reported to possess potent and targeted subcellular modulatory effects on EMT, particularly the inhibition of EMT-promoting proteins (vimentin, slug) and simultaneous promotion of the epithelial protein, E-cadherin (Buhrmann et al., 2015), thus showing an inhibitory effect on the highly metastatic tumors.

Resveratrol has also been announced to make use of integrins as both, surface receptors and signaling molecules, for exerting its anti-tumor effects (Lin et al., 2006; Belleri et al., 2008; Varoni et al., 2016). Integrin families are heterodimeric cell adhesion and signaling molecules that play a central role as signal transducers in the interaction between cells and extracellular matrix (Shakibaei et al., 1997; Shakibaei, 1998; Shakibaei and Merker, 1999; Mueller et al., 2022), thus display a crucial role in the differentiation and function of cells in healthy tissues (Carter et al., 1990; Chen, 1990; Shakibaei, 1995). Moreover, these specific compounds are remarkably up-regulated in cancer cells, what is associated with increased risk for tumor migration and metastasis (Huang et al., 2021). Researchers aim to take advantage of this property and thus are investigating the chance of using integrins as tumor markers (Jones et al., 1992). Specifically for colorectal carcinoma, α1-integrin has already been shown to be associated with increased tumorigenesis, CRC cell migration and invasion (Li et al., 2020), and β6-integrin provided even more accurate prognosis than the already established tumor marker carcinoembryonic antigen (CEA) for tumor surveillance of patients with advanced CRC stage (Bengs et al., 2019). Furthermore, there are indications that inhibition of αvβ6-integrin could reduce the risk of liver metastasis in diabetic CRC patients (Wang et al., 2021) and that resveratrol-binding αvβ3-integrin inhibits tumor growth and metastasis as promising target for cancer therapy (Cheng et al., 2021; Chen et al., 2022). Moreover, down-regulation of focal adhesion kinase (FAK), a specific subcellular target protein of integrins (Lipfert et al., 1992) that is also of great importance in cell migration and invasion (Buhrmann et al., 2017), represents an important mechanism here (Cheng et al., 2021) and resveratrol modulates FAK phosphorylation (Buhrmann et al., 2017).

Recently, we have demonstrated that resveratrol shows one of its important anti-proliferative and -viable properties *via* modulation of the β1-integrin pathway by rearrangement of β1-integrin receptors to use them for signal transduction as well as for exertion of resveratrol’s proliferation- and viability-inhibitory capabilities (Brockmueller et al., 2022) in CRC cells.

In the present work, we address the question of whether and how resveratrol can affect the invasion and metastasis potential and thus the EMT of CRC cells *via* β1-integrin axis. Within this study, implications of β1-integrin-SO/ASO (β1-SO/ASO) and NF-κB-SO/ASO in HCT116 and RKO CRC cells were compared in a multicellular, pro-inflammatory TME *in vitro.* This 3D-tumor cultures, composed of cancer cells, T-lymphocytes and fibroblasts have been established throughout our previous studies (Buhrmann et al., 2020; Brockmueller et al., 2021) as well as in other research groups (Gao et al., 2021).

## Material and methods

### Antibodies and reagents

Monoclonal antibodies to NF-κB (#MAB5078), and phospho-specific p65-NF-κB (#MAB7226), MMP-9 (#MAB911), polyclonal caspase-3 (#AF835) were purchased from R&D Systems (Heidelberg, Germany). Monoclonal antibodies to anti-FAK (#610088), anti-phospho-FAK (#558540) were from Becton Dickinson (Heidelberg, Germany). Monoclonal antibodies to β-actin (#A4700), resveratrol, alginate, DAPI, Fluoromount were from Sigma-Aldrich (Taufkirchen, Germany). Monoclonal anti-E-cadherin (#sc-21791), anti-vimentin (#sc-53464), anti-slug (#sc-166476), anti-paxillin (#sc-365059) normal mouse IgG (#sc-2025) were from Santa Cruz Biotechnology (Dallas, Texas, United States). Monoclonal anti-β1-integrin (#14-0299-82) and anti-CXCR4 (#35-8800) were from Thermo Fisher Scientific (Langenselbold, Germany). Secondary rhodamine-coupled antibodies for immunofluorescence were from Dianova (Hamburg, Germany), and alkaline phosphatase-linked antibodies for Western blotting were from EMD Millipore (Schwalbach, Germany). Resveratrol was prepared in 100 mM stocks with ethanol and directly diluted in the cell culture medium for CRC cell treatment, without exceeding an ethanol concentration of 0.1% during the investigations.

### Cells and preparation

For the presented studies, two different human CRC cell lines [HCT116 from European Collection of Cell Cultures (Salisbury, United Kingdom) and RKO from American Type Culture Collection (Manassas, Virginia, United States)], human T-lymphocytes [Jurkat from DSMZ-German Collection of Microorganisms and Cell Cultures (Braunschweig, Germany)] and human fibroblasts [MRC-5 from European Collection of Cell Cultures (Salisbury, United Kingdom)] were used. The cell culture setting and preparation corresponds to the one already described earlier (Buhrmann et al., 2020; Brockmueller et al., 2022). 3% fetal bovine serum (FBS) or 10% FBS, 1% glutamine, 1% penicillin/streptomycin solution (10.000 IU/10.000 IU), 75 μg/ml ascorbic acid, 1% essential amino acids and 0.5% amphotericin B solution were added to Dulbecco’s modified Eagle’s medium/F-12 from Sigma-Aldrich (Taufkirchen, Germany) and used as cell culture medium.

### Transfection

To study the effects of transfection, 0.5 µM antisense oligonucleotides (ASO) or control sense oligonucleotides (SO) was incubated in Lipofectin transfection reagent (Invitrogen, Karlsruhe, Germany) and added into the experimental well-plate. Oligonucleotides from Eurofins MWG Operon (Ebersberg, Germany) were modified with phosphonothioate to preserve the oligonucleotides from cell nucleases, the exact procedure has already been described (Buhrmann et al., 2016; Buhrmann et al., 2020), and used for transient transfection with antisense/sense oligonucleotides (ASO/SO) based on β1-integrin or p65-NF-κB:

a) β1-integrin-ASO (5′-TAG​TTG​GGG​TTG​CAC​TCA​CAC​A-3′),
b) β1-integrin-SO (5′-TGT​GTG​AGT​GCA​ACC​CCA​ACT​A-3′),
c) NF-κB/p65-subunit-ASO (5′-gGA​GAT​GCG​CAC​TGT​CCC​TGG​TC-3′),
d) NF-κB/p65-subunit-SO (5′-gAC​CAG​GGA​CAG​TGC​GCA​TCT​C-3′).

### 3D-alginate tumor culture *in vitro*

In the current investigations, the *in vivo* condition of a cancerous patient was simulated by a 3D-alginate culture model *in vitro*. This enables an animal-free investigation of resveratrol’s anti-tumor effect in a pro-inflammatory tumor microenvironment. The present study focuses on the exploration of resveratrol’s β1-integrin-mediated effects on the migration and invasion of CRC cells using β1-integrin-ASO/SO as well as NF-κB-ASO/SO for comparison.

The experiments were carried out with CRC-alginate balls comprising an average size of 4 mm which were produced as described in numerous publications of our research group (Buhrmann et al., 2020; Brockmueller et al., 2022). Then they were assembled in a composition of MRC-5 fibroblasts as monolayer on the bottom of 12-well-plates, and Jurkat T-lymphocytes floating in the cell culture medium, which has been established as a suitable TME simulation for other cancer cells as well (Gao et al., 2021). In the present work, the alginate balls were made from HCT116 or RKO cells in 12-well-plates and the alginate coating ensures that the CRC cells do not mix with the other cell types during the experiments. A control without TME (Ba. Co.) and a TME control (TME Co.) without treatment additives were established. The treated cells received resveratrol (1, 5 µM), β1-integrin-ASO/SO (0.5 µM), NF-κB-ASO/SO (0.5 µM) or a combination thereof as supplements to 3% FBS (serum-starved) cell culture medium with a running time of 10–14 days for all samples.

### Three invasion attempts

To observe the invasion and to draw conclusions about CRC’s metastatic properties, HCT116 and RKO were treated as explained before (Ba. Co., TME Co., resveratrol, β1-integrin-SO/ASO, NF-κB-SO/ASO) for 10–14 days and evaluated using three different invasion assays:

A) Firstly, the bottom of 12-well-plates was photographed in phase contrast with a Zeiss Axiovert 40 CFL microscope (Oberkochen, Germany) in order to compare the CRC cell colonies that had settled. To determine the average size of settled colonies, 25 colonies of each treatment were measured.
B) Secondly, the 12-well-plates were fixated with Karnovsky solution for 30 min, stained with toluidine blue. To determine the average colony, count of stained colonies, labelled as invasion, colonies were counted from three wells at each treatment.
C) Thirdly, the experiment was set up with identical treatments in 6-well-plates. As a special feature, a square cover glass was placed in each well on which the migrating CRC cell colonies settled. The glass slides were fixated with methanol for 30 min, then rinsed with Hank’s solution (3 times), incubated for 15 min in the dark with DAPI and covered in Fluoromount for photographic analysis using a Leica DM 2000 microscope (Wetzlar, Germany).

### Immunofluorescence investigation

Leica (Wetzlar, Germany) DM 2000 microscope with LAS V4.12 software was used for the generation of immunofluorescence images. For preparation, 6000 CRC cells (HCT116 or RKO) were sown on a small round glass plate. After 24 h, the TME was recreated in a modified manner by placing the small glass plates on a meshed bridge in a 6-well-plate containing fibroblasts as monolayer, floating T-lymphocytes and reagents according to the treatment pattern described above. After 1 day, the glass slides were removed from well-plates and washed with Hank’s salt solution three times, ensuring that only CRC cells were left for immunolabelling. Then, CRC-glass plates were fixated in methanol for 30 min and prepared for immunofluorescence microscopy as described in detail before (Brockmueller et al., 2022; Mueller et al., 2022). In the present work, CRC cells were immunolabeled with E-cadherin-, NF-κB- paxillin- or slug-antibodies. The primary antibody dilution was 1:80 and the secondary antibody dilution was 1:100 each. To reveal the cell nuclei, each slide was stained with DAPI for 15 min and then fixated with Fluoromount.

### Immunoblotting

For Western blot analysis, HCT116 or RKO 3D-alginate balls were treated with the aforementioned substances. After 10–14 days, the alginate balls were removed from 12-well-plates with bent tweezers, transferred to 12-well-plates containing Hank’s salt solution, and washed on a gentle waving shaker. This procedure was repeated at least three times, ensuring that no T-lymphocytes adhered to the CRC-alginate balls which was verified by observation with a phase contrast microscope. Then, the clean CRC-alginate balls were dissolved in sodium citrate (55 mM) for 30 min to isolate CRC cells which were resuspended in lysis buffer as described before (Brockmueller et al., 2022). After 30 min of centrifugation, the liquid supernatant was kept frozen (−80°C) and samples were further processed with a Bio-Rad (Munich, Germany) transblot apparatus for densitometric evaluation with Bio-Rad Quantity One analysis software as explained in detail in our earlier work (Brockmueller et al., 2022; Mueller et al., 2022). In the current study, the beforementioned antibodies were used in 1:10.000 dilution and β-actin served as loading control.

### Statistical analysis

We performed all assays as three independent repetitions and used an unpaired student’s t-test for statistical analysis. Results matched by one-way ANOVA followed by post hoc test to compare parameters within the groups. At the outcomes, a *p*-value less than 0.05 was considered as statistically significant.

## Results

The aim of our study was to determine the role of β1-integrin receptors in the anti-invasive, anti-metastatic and anti-inflammatory effects of resveratrol in two CRC cell lines (HCT116, RKO) in a pro-inflammatory, multicellular, *in vivo*-like tumor microenvironment *in vitro* by β1-integrin knockdown and NF-κB knockdown *via* antisense oligonucleotides (ASO).

### Resveratrol targets the β1-integrin receptors to promote TME-down-regulated E-cadherin expression and suppress up-regulated paxillin expression in CRC cells

**FIGURE 1:**
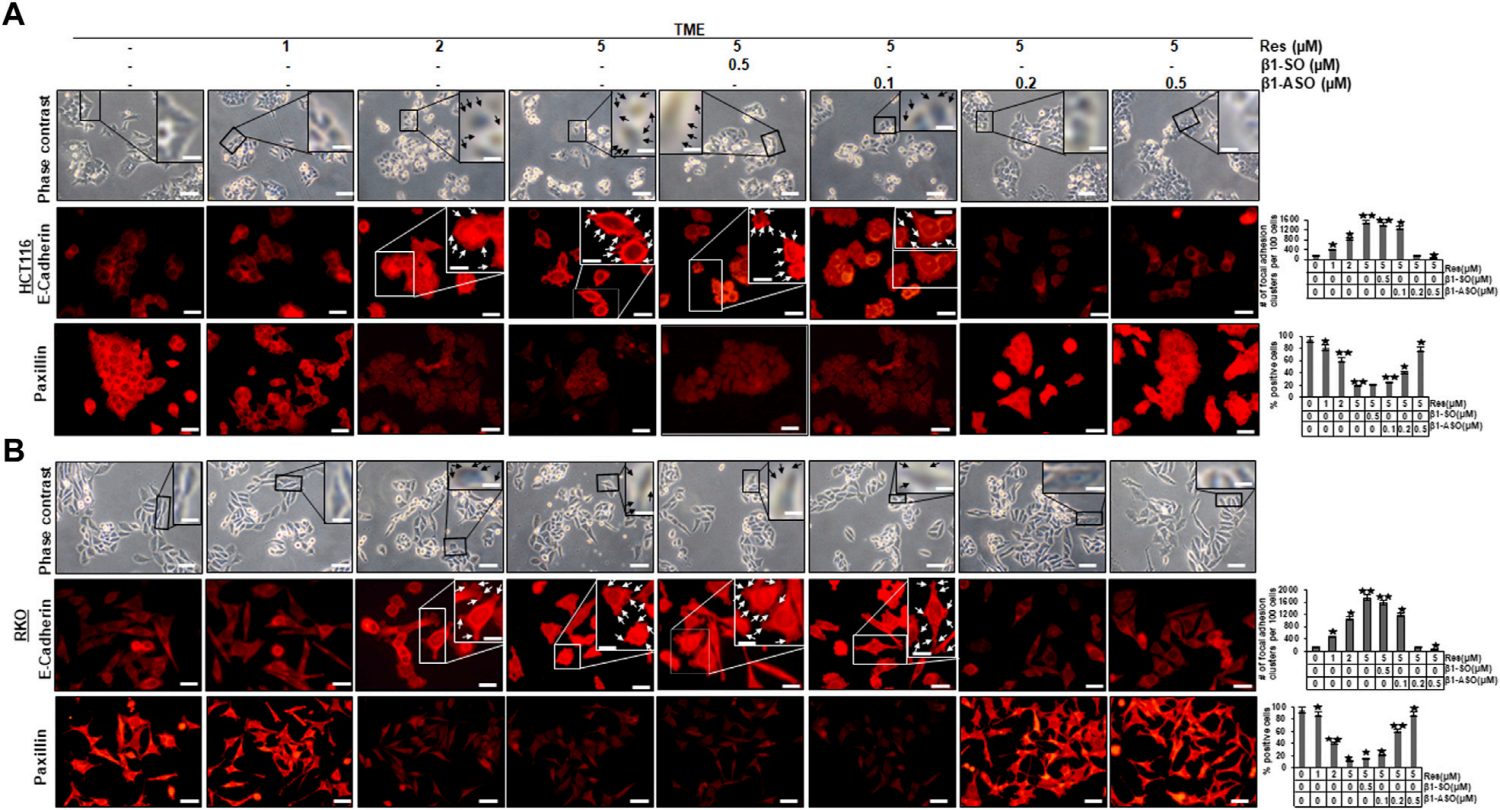
Impact of resveratrol or/and ASO against β1-integrin on TME-mediated cell morphology and expression of E-cadherin and paxillin in CRC cells. HCT116 **(A)** and RKO **(B)** cells were grown on coverslips in TME, left untreated or treated with increasing doses of resveratrol (1, 2, 5 µM) or were transfected with β1-SO or β1-ASO as outlined in Material and Methods. After rinsing with phosphate-buffered water, the cell morphology of CRC cells was examined by phase contrast **(A,B)**, (top row each**)**. Immunolabeling (red) with anti-E-cadherin **(A,B)**, (middle row each**)** or paxillin **(A,B)**, (bottom row each) was observed by immunofluorescence microscopy. Special attention is paid to the discovered resveratrol-induced plaque-like deposits, highlighted with black **(A,B)**, (top row each) and white **(A,B)**, (middle row each) arrows. Statistical analysis: Plaque-like deposits were quantified by couting in 20 different microscopiy fields. Related to TME control, *p < 0.05 and **p < 0.01. Microscope: Leica DM 2000. Magnification ×600; scale bar = 30 µm. Inset magnification: ×1200; scale bar = 15 µm.

Investigations on CRC primary tumors showed that they were prone to have a dedifferentiated, mesenchymal phenotype, a noticeably reduced E-cadherin expression, an increased paxillin expression, and a high tendency to metastasize (Kaihara et al., 2003; Buhrmann et al., 2015; Wen et al., 2020). Therefore, we examined HCT116 and RKO cells, grown on glass slides, in more detail using the phase contrast microscope, and observed that the HCT116 and RKO control cultures exhibited evenly distributed cell colonies with a slightly epithelial morphology, smooth surface, and close cell to cell contacts (Figures 1A,B), similar to treatment with only β1-integrin-SO or -ASO (β1-SO or -ASO). In contrast, the RKO control cells showed a more fibroblast-like to mesenchymal morphology, less colony formation, and fewer cell to cell contacts. Interestingly, treatment of both CRC cells with resveratrol resulted in 1) increased cell to cell contact (epithelial shape) and a rounder shape of CRC cells though and 2) the development of small conspicuous plaque-like deposits of the cell membrane on HCT116 (Figure 1A, top row, black arrows) and RKO cells (Figure 1B, top row, black arrows) in a dose-dependent (1, 2, 5 µM resveratrol) manner, similar to the combined treatment with resveratrol (5 µM) and/or β1-SO (0.5 µM). Even more interestingly, treatment with resveratrol and β1-ASO significantly decreased or resolved the development of small conspicuous plaque-like deposits of the cell membrane on HCT116 (Figure 1A, top row) and RKO cells (Figure 1B, top row) in a dose-dependent (0.1, 0.2, 0.5 µM β1-ASO) manner in both cell lines. Together, these findings indicate that the detected resveratrol-induced plaque formation in CRC cells by targeting β1-integrin receptors was related to an increased E-cadherin expression, leading to a more stable local adhesion and therefore rather epithelially shape. We wondered whether this resveratrol-induced plasma membrane plaques contain the biomarker for epithelial phenotype, E-cadherin, and therefore examined its expression by immunofluorescence analysis (Figures 1A,B; middle row each). The untreated CRC cells in TME showed minimal E-cadherin expression on the surface, similar to treatment with β1-SO (0.5 µM) or β1-ASO (0.5 µM) alone. On the other hand, the expression of E-cadherin on the surface of CRC cells treated with resveratrol (1, 2, 5 µM) alone showed a significant increase in E-cadherin expression in a concentration-dependent manner, which was regulated similar to cells treated with β1-SO and resveratrol in combination (Figures 1A,B; middle row each, white arrows). In contrast, knockdown of β1-integrin with β1-ASO in a concentration-dependent (0.1, 0.2, 0.5 µM) manner abolished the blocking effect of resveratrol on the expression of the above-mentioned epithelial biomarkers. To further investigate the impact of the β1-integrin-mediated effect of resveratrol on EMT processes in CRC cells, we also immunolabelled HCT116 as well as RKO cells with an antibody against the cell migration marker paxillin (Figures 1A,B; bottom row each). Confirming previous results, both CRC cell lines showed high paxillin expression in the untreated TME control, which decreased significantly with increasing resveratrol (1, 2, 5 µM) concentration. The largely suppressed expression of paxillin by resveratrol (5 µM) in CRC cells remained low even with combined treatment of the CRC cells with 0.5 µM β1-SO. However when β1-ASO (0.1, 0.2, 0.5 µM) was added to resveratrol-treated HCT116 or RKO cells, paxillin expression became increasingly intense. In summary, these results indicate a strong migration inhibitory effect of resveratrol on CRC cells (Figures 1A,B) and the important role of β1-integrin receptors in resveratrol-enhancing anti-tumor effect on CRC cells in TME, whereby these effects were not cell line specific.

### Resveratrol targets β1-integrin receptors and suppresses TME-up-regulated phosphorylation and nuclear NF-κB translocation in CRC cells

**FIGURE 2:**
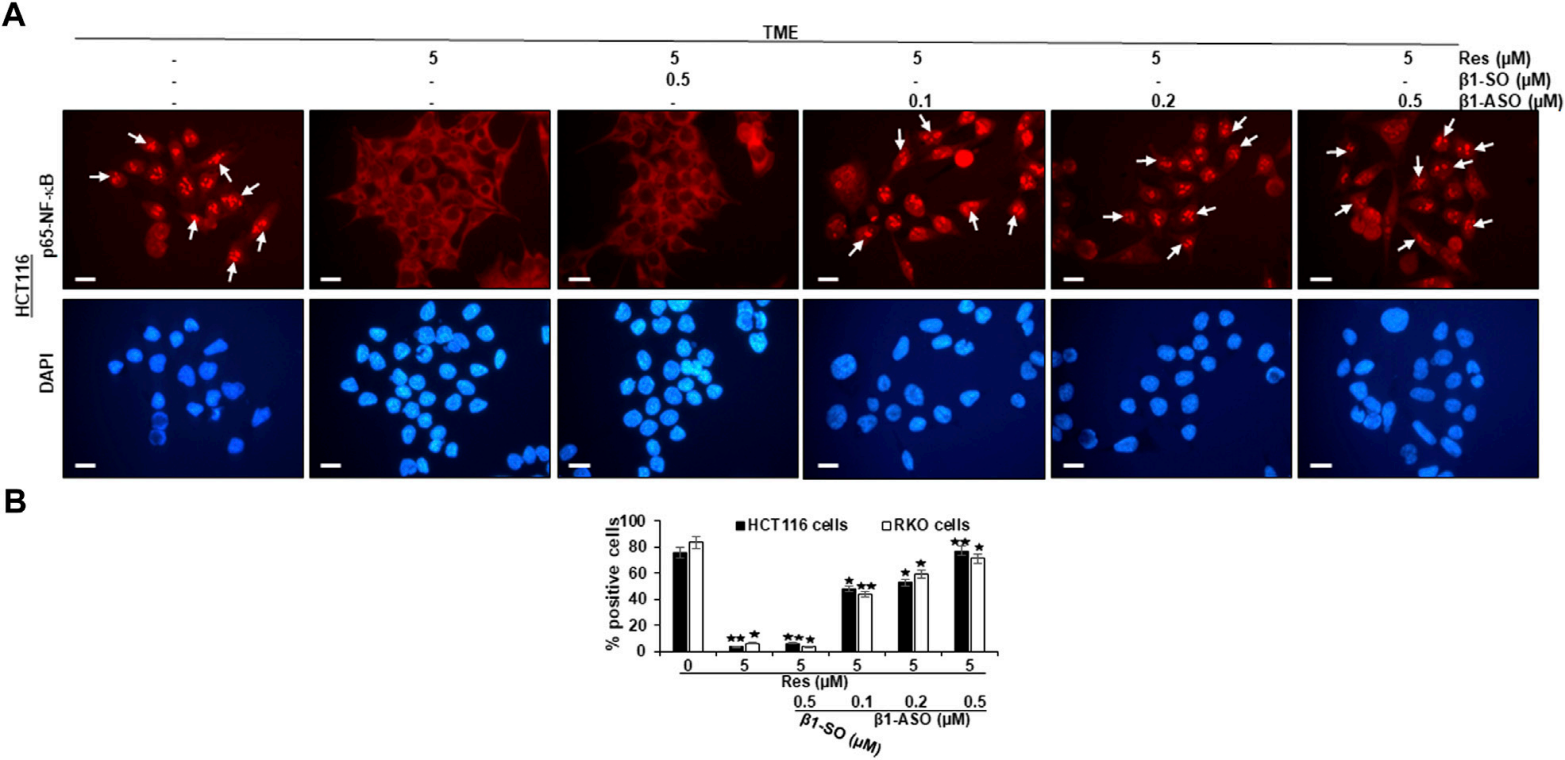
Impact of resveratrol or/and ASO against β1-integrin on TME-mediated activation and nuclear translocation of p65-NF-κB in CRC cells. HCT116 **(A,B)** and RKO **(B)** cells in TME on cover glasses without treatment or only treated with resveratrol (5 µM) or resveratrol-treated (5 µM) with addition of β1-SO (0.5 µM) or knocked down with β1-ASO (0.1, 0.2, 0.5 µM). Anti-phosho-NF-κB immunolabeled (red) and DAPI-stained (blue). White arrows = p65-NF-κB positive CRC cells. Microscope: Leica DM 2000. Magnification ×600; scale bar = 30 µm. Statistical evaluation: *p < 0.05 and **p < 0.01, compared to TME control.

As resveratrol is known to be a compound that inhibits inflammation in CRC cells (Buhrmann et al., 2016), we wanted to examine its influence on NF-κB activation and furthermore the role of β1-integrin receptors in this context. For this purpose, CRC cells (HCT116, RKO) were cultured on round glass coverslips and examined by immunofluorescence microscopy as described in the Material and Methods section. Here, HCT116 cells in untreated TME (TME control) showed a distinct, luminescent immunolabeling (Figure 2A, exemplary marked with white arrows) as a sign of high, inflammation-induced NF-κB phosphorylation and nuclear translocation in the majority of CRC cells (Figures 2A,B) and confirmatory, this expression was similar to TME-HCT116 cells treated with β1-SO (0.5 µM) or β1-ASO (0.5 µM) alone. Moreover, treatment with resveratrol (5 µM) led to impressive changes in TME grown CRC cells with a significant down-regulation of NF-κB phosphorylation and nuclear translocation compared to the TME control, resulting in barely labeled, pale cell nuclei (Figure 2A). This effect remained the same with β1-SO (0.5 µM) addition to resveratrol-treated HCT116 cells in TME, validating β1-SO as suitable control substance (Figures 2A,B). However, when β1-ASO (0.1, 0.2, 0.5 µM) was added to resveratrol-treated TME cultures for the purpose of β1-integrin knockdown, a concentration-dependent increase of phosphorylation and nuclear translocation of NF-κB was found in CRC cell nuclei, due to the gradual removal of resveratrol’s effect (Figures 2A,B). Interestingly, the statistical analysis of RKO CRC cells undergoing same treatments (Figure 2B) confirmed our observations made on HCT116 (Figures 2A,B), leading to the assumption that these are transferable to other CRC cell lines, whereas DAPI staining verified the vitality of the photographed CRC cells by blue DNA labeling. Altogether, these results demonstrate a strong, non-cell line specific anti-inflammatory effect of resveratrol and furthermore suggest a significant limitation of resveratrol’s anti-inflammatory impact by β1-integrin knockdown.

### Resveratrol targets β1-integrin receptors and suppresses TME-induced activation of EMT-related transcription factor (slug) in CRC cells

**FIGURE 3:**
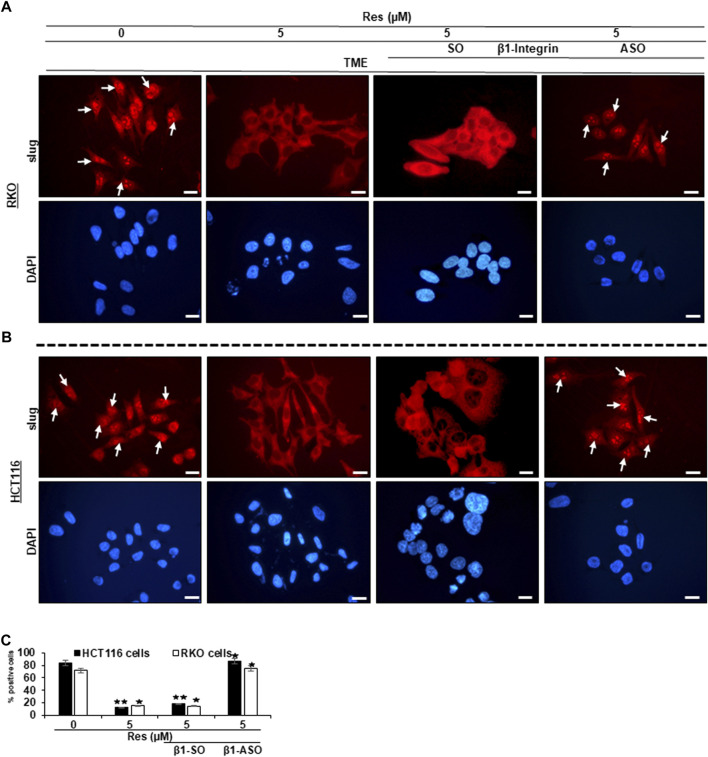
Impact of resveratrol or/and ASO against β1-integrin on TME-mediated EMT-linked transcription factor slug activation in CRC cells. RKO **(A,C)** and HCT116 **(B,C)** cells in TME on coverslips, left untreated or treated with resveratrol (5 µM) or resveratrol-treated (5 µM) with addition of β1-SO (0.5 µM) or knocked down with β1-ASO (0.5 µM). CRC cells were immunolabeled with anti-slug (red) and DAPI-stained (blue). White arrows = slug positive CRC cells. Microscope: Leica DM 2000. Magnification ×600; scale bar = 30 µm. Statistics: *p < 0.05 and **p < 0.01, in relation to TME control.

Since we have already found out that a pro-inflammatory, multicellular TME promotes EMT and resveratrol is able to intervene this conversion (Buhrmann et al., 2015), we were then interested in the extend of major EMT transcription factor slug expression, the influence of resveratrol and especially the role of β1-integrin receptors in this process. In Figure 3, RKO (Figure 3A) and HCT116 are shown (Figure 3B), which were grown on small cover glasses in TME, subsequently immune-marked with anti-slug antibody and analyzed by immunofluorescence microscopy as described in the Material and Methods section. We observed same significant expression tendencies in both cell lines, RKO and HCT116: In the TME (TME control), the majority of CRC cells showed cell nuclei with bright, slug-positive labeling, exemplary highlighted with white arrows (Figures 3A,B), whereas β1-SO (0.5 µM) or β1-ASO (0.5 µM) addition had no visible influence. However, resveratrol-treatment (5 µM), remarkably suppressed slug expression in the TME compared to TME control, leading to pale labelled inconspicuous cell nuclei. Interestingly, addition of 5 µM resveratrol to β1-SO (0.5 µM) treated CRC cells did not change this expression pattern, leading to confirmation of β1-SO as suitable control substance (Figures 3A,B). In contrast, in the combined treatment consisting of 5 µM resveratrol and 0.5 µM β1-ASO, there were almost as many slug-positive labeled CRC cells found as in the TME control (Figures 3A,B), underscoring that resveratrol was not able to fully exert its EMT-inhibitory effect when β1-integrin was knocked down. The direct statistical comparison of RKO and HCT116 (Figure 3C) proved the described non-cell line specific effects. To ensure the viability of evaluated CRC cells, supplementary DAPI staining (blue) was performed (Figures 3A,B). In summary, these results highlight the significant EMT-inhibitory and reproducible effect of resveratrol in the two CRC cell lines RKO and HCT116, and point out the major role of β1-integrin receptors in mediating these effects.

### Resveratrol-promoted repression of TME-induced migration and invasion, similarly to knockdown of NF-κB, is blocked by knockdown of β1-integrin in CRC cells

**FIGURE 4:**
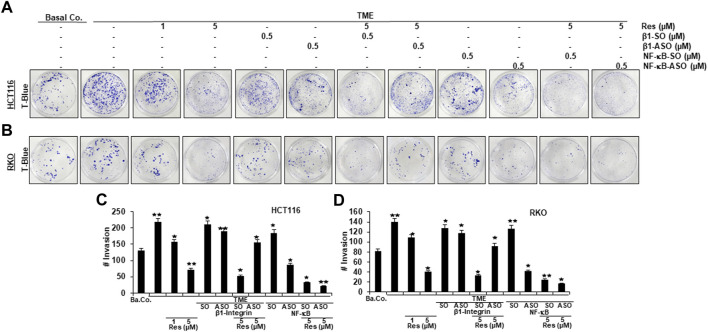
Impact of resveratrol or/and ASO against β1-integrin or against NF-κB on TME-promoted CRC cell invasion in alginate cultures. Serum-starved HCT116 **(A)** and RKO **(B)** cells, cultured in 3D-alginate, emigrated under following treatments: untreated (Basal Co.), TME control, resveratrol (1, 5 µM), β1-SO/ASO (0.5 µM), NF-κB-SO/ASO (0.5 µM) or co-treatment of sense or antisense oligonucleotides with resveratrol (5 µM). CRC cells were stained with toluidine blue (T-Blue) after settling on the bottom of 12-well-plates. Statistical evaluation: Compared to TME control, *p < 0.05 and **p < 0.01 for HCT116 **(C)** and RKO **(D)**.

**FIGURE 5:**
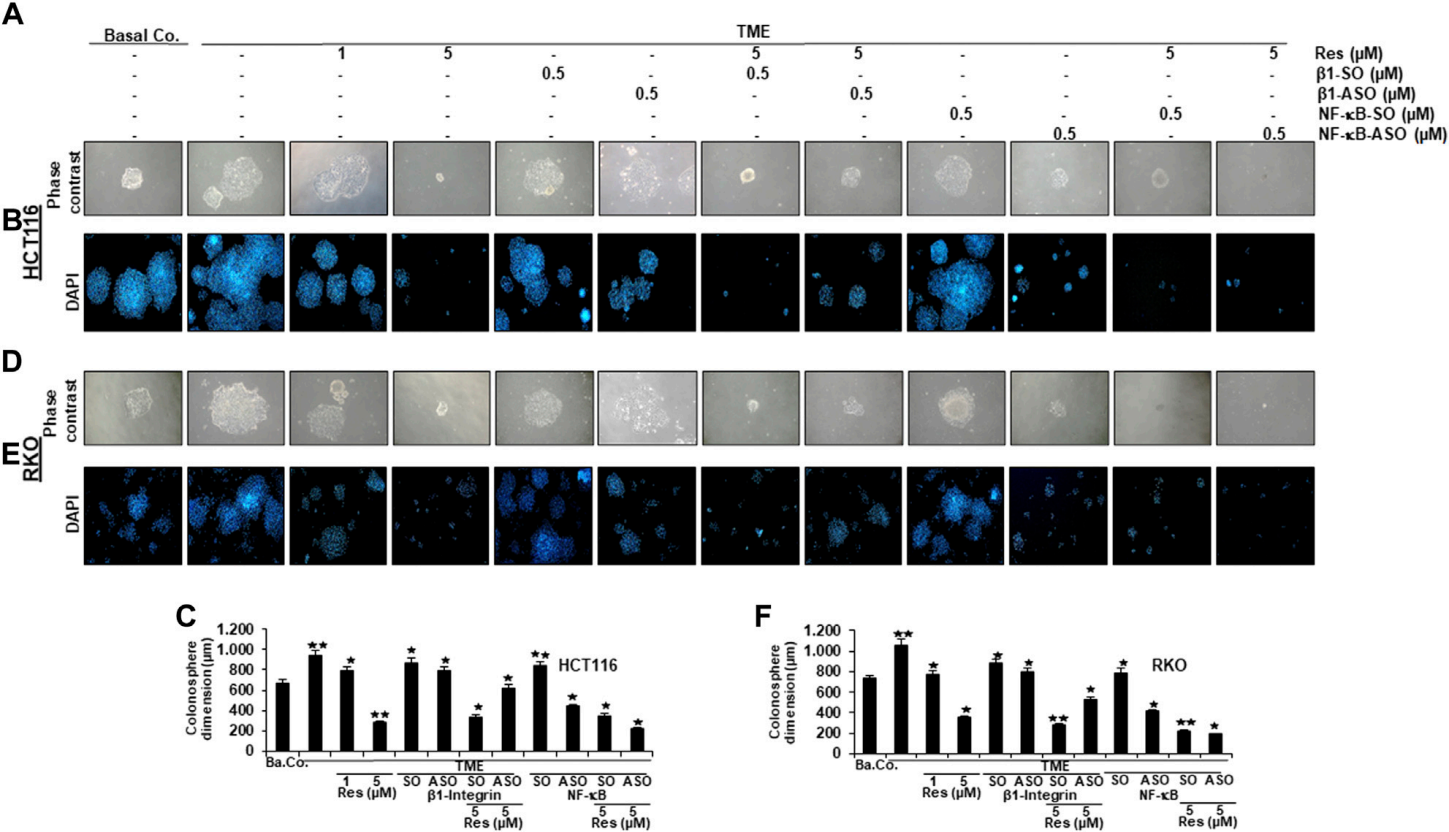
Impact of resveratrol or/and ASO against β1-integrin or against NF-κB on TME-promoted CRC cell metastasis and formation of colonospheres in alginate cultures. Serum-starved HCT116 **(A,B)** and RKO **(D,E)** cells from 3D-alginate, settled on square cover glasses as colonies under different treatment conditions: untreated (Basal Co.), TME control, resveratrol (1, 5 µM), β1-SO/ASO (0.5 µM), NF-κB-SO/ASO (0.5 µM) or combination of SO/ASO (0.5 µM) and resveratrol (5 µM). CRC cells were photographed using phase contrast (magnification ×100, Zeiss Axiovert 40 CFL microscope) and DAPI-staining (magnification ×50, Leica DM 2000 microscope). Statistical analysis for HCT116 **(C)** and RKO **(F)**: Relative to TME control, *p < 0.05 and **p < 0.01.

In order to further investigate the role of β1-integrin receptors in mediating the anti-metastatic and anti-invasive effects of resveratrol, we carried out three invasion assays of HCT116 (Figures 4A,C, Figures 5A–C) and RKO (Figures 4B,D, Figures 5D–F) as described in the Material and Methods section. For this purpose, β1-integrin was knocked down *via* β1-ASO and because of its important role in inflammation and tumor progression (Rasool et al., 2021), NF-κB was knocked down as well using NF-κB-ASO.

The focus of our invasion assays was the observation of spheres of CRC cells that have been migrated from 3D-alginate beads, which represent an *in vivo*-like simulation of metastasis and invasion in an *in vitro* culture model. The emigrated CRC-colonies that had attached to the bottom of well-plates were stained with toluidine blue (Figure 4), whereas colonies that had settled on square cover glasses were analyzed by phase contrast (Figures 5A,D) or additionally stained with the before mentioned DAPI-method (Figures 5B,E), in order to demonstrate the vitality of CRC cells. Cross-methodology, based on invasion assays and statistical analysis, showed that the pro-inflammatory, multicellular TME increased both, the number of invaded colonies and the average diameter of colonies compared to the baseline control (Ba.Co.) without fibroblasts and T-lymphocytes (Figures 4A,B Figures 5A,B,D,E). The administration of β1-SO (0.5 µM) or β1-ASO (0.5 µM) alone had no significant modifying effects on the CRC-colonies in TME. Apart from that, a concentration-dependent reduction of invaded colonies and colony size by resveratrol (1, 5 µM) treatment in the TME was clearly visible, and could also be observed in concomitant treatment of resveratrol (5 µM) together with β1-SO (0.5 µM), validating β1-SO as an appropriate control agent. However, the knockdown of β1-integrin *via* β1-ASO strongly reduced the inhibitory effect of resveratrol, barely affecting colony size and number of migrated CRC cells. Even TME-treatment using a lower concentration of resveratrol (1 µM) and without β1-integrin knockdown, did show greater effects (Figures 4A,B, Figures 5A,B,D,E).

Simultaneously, resveratrol (5 µM), comparable to the knockdown of NF-κB (0.5 µM NF-κB-ASO), significantly reduced the number of CRC cell migration and their average size, highlighting resveratrol’s anti-invasive effect and its ability to even intensify the inhibitory effect of NF-κB-ASO (0.5 µM). Moreover, it was found that there was no significant difference between co-treatment of resveratrol (5 µM) and NF-κB-SO (0.5 µM) or resveratrol (5 µM) alone on reduced inhibition of migration and invasion and their size on CRC cells (Figures 4A,B, Figures 5A,B,D,E). These findings were further supported by the quantification of colonosphere formation, migrated CRC cells and their size. All of the described results were reproducible in both cell lines, HCT116 and RKO, indicating a non-cell line specific effect. Altogether, these findings further support the idea that resveratrol uses β1-integrin receptors to repress the pro-inflammatory TME-induced migration, invasion and signaling pathway of CRC cells.

### Resveratrol targets β1-integrin receptors to block TME-induced expression of metastasis-related factors and p65-NF-κB phosphorylation in CRC cells

To screen changes in protein expressions and associated modulation of various signaling pathways, we carried out extensive Western blot analyses. Samples were obtained from CRC cells (HCT116 or RKO) grown as alginate bead cultures and treated as described in Material and Methods for 10–14 days.

**FIGURE 6:**
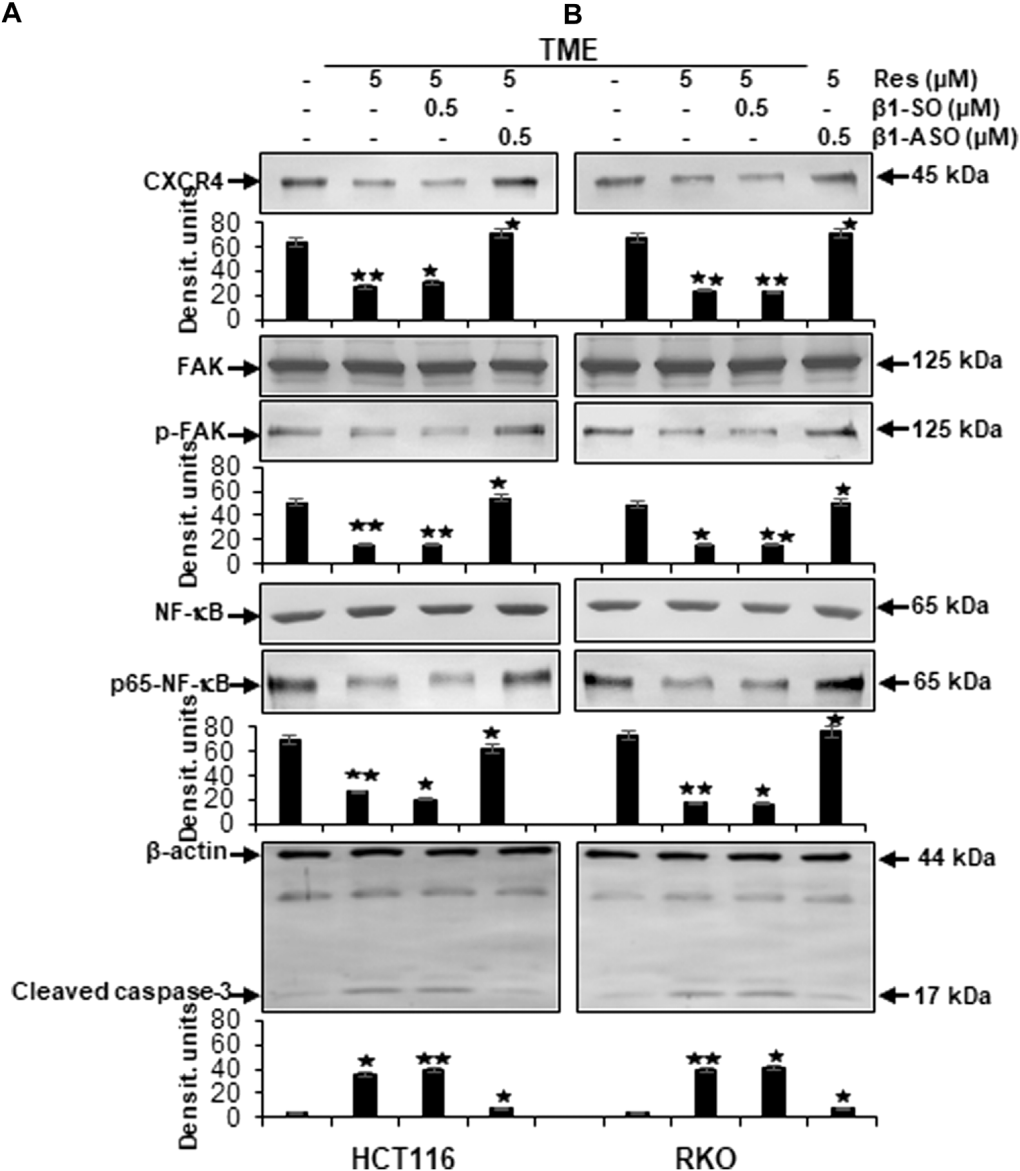
Impact of resveratrol or/and ASO against β1-integrin on TME-promoted activation of metastasis and apoptosis parameters in CRC cells. HCT116 **(A)** and RKO **(B)** derived from 3D-alginate cultures were grown untreated or treated with 5 µM resveratrol alone or in combination with 0.5 µM β1-SO or 0.5 µM β1-ASO (*x*-axis) and probed with antibodies against CXCR4, FAK, p-FAK, NF-κB, p65-NF-κB and cleaved caspase-3. Loading control: β-actin. Densitometric units complementing Western blot results (*y*-axis). For densitometric analysis, data were compared to TME control: *p < 0.05 and **p < 0.01.

At first, we found that the metastasis parameters CXCR4 and phosphorylated FAK as well as inflammation parameter phosphorylated NF-κB were highly expressed in HCT116 or RKO cells grown in TME without any treatment. Interestingly, compared to TME control, same biomarkers were significantly down-regulated by resveratrol (5 µM) in TME-cultures, similar to β1-SO-cultures (0.5 µM) co-treated with 5 µM resveratrol, (Figure 6). Contrarily, in CRC cultures with β1-integrin knockdown (0.5 µM β1-ASO), no suppression of CXCR4, phosphorylated FAK and phosphorylated NF-κB could be observed by resveratrol co-treatment (5 µM) so that their expression pattern resembled the TME control (Figure 6). In addition, cultures solely treated with resveratrol (5 µM) or with resveratrol (5 µM) in combination with β1-SO (0.5 µM) clearly showed increased apoptosis, manifested by a high caspase-3 level in the TME compared to a low apoptosis rate in the untreated TME control. In β1-integrin knockdown (0.5 µM β1-ASO) cultures, however, resveratrol’s anti-apoptotic effect could not be detected and a low apoptosis rate was observed again (Figure 6). Our results were supported by the consistent expression of non-phosphorylated FAK and NF-κB in all of the treatments, serving as reference control, and consistent expression of β-actin serving as loading control (Figure 6).

**FIGURE 7:**
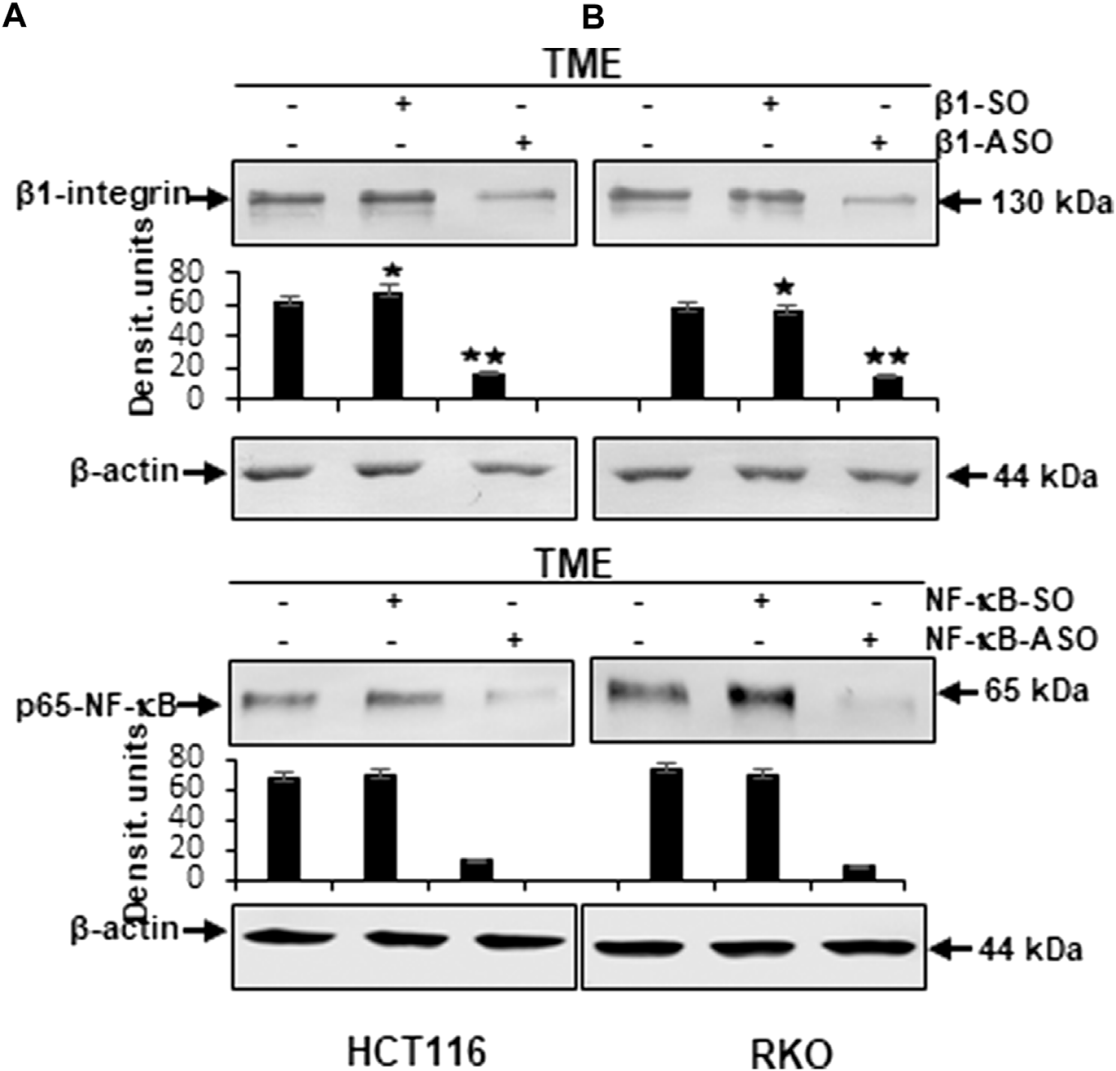
Western blot investigation on the efficacy of β1-integrin and NF-κB knockdown by antisense oligonucleotides in CRC cells. *X*-axis: HCT116 **(A)** and RKO **(B)** samples from 3D-alginate TME were untreated or treated with β1-SO/NF-κB-SO or β1-ASO/NF-κB-ASO (0.5 µM). Immunoblotting with anti-β1-integrin or with anti-p-65-NF-κB and β-actin as a loading control. *Y*-axis: Densitometric units (*y*-axis) complementing Western blot results. Statistical analysis: *p < 0.05 and **p < 0.01, comparison to TME control.

Furthermore, to reassure the effectiveness of β1-integrin knockdown (β1-ASO) and control substance (β1-SO), or NF-κB knockdown (NF-κB-ASO) and the control substance (NF-κB-SO), we also performed immunoblots with anti-β1-integrin or with anti-p65-NF-κB that confirmed the chosen dosages (0.5 µM β1- and NF-κB-SO/ASO) in both cell lines (HCT116 and RKO), by demonstrating significant β1-integrin or NF-κB down-regulation of β1-ASO or NF-κB-ASO treated CRC cells, respectively, whereas β1-integrin and NF-κB were up-regulated in untreated TME or TME-CRC cells treated with β1-SO or NF-κB-SO (Figures 7A,B). These results, supporting our assumption that resveratrol uses β1-integrin receptors and NF-κB transcription factor to inhibit metastasis in CRC cells, encouraged us to further investigate the level of EMT-protein expression.

### Resveratrol modulates TME-triggered inflammation, EMT, invasion, and TME-suppressed apoptosis and acts synergistically with NF-κB-ASO in CRC cells but not by knockdown of β1-integrin in CRC cells

Whether and by which pathway resveratrol can modulate TME-induced EMT, invasion, migration as well as upregulation of NF-κB, NF-κB-dependent inflammation and apoptosis, was investigated by using HCT116 and RKO CRC cells. Analogous to previously explained invasion assays (Figure 4), cells were left untreated in alginate beads as a baseline control (Ba.Co.) or in TME (TME control) or treated with different concentrations of resveratrol (1, 5 µM) or with NF-κB-SO/-ASO (0.5 µM) or with β1- SO/-ASO (0.5 µM) or with a combination of resveratrol (5 µM) and NF-κB-SO/-ASO (0.5 µM) or β1-SO/-ASO (0.5 µM) for 10–14 days as described in Methods.

### Resveratrol targets β1-integrin and blocks TME-induced expression of EMT-related biomarkers in the same way as NF-κB-ASO in CRC cells

Initially, with Western blot studies of previously described cell samples we aimed to visualize EMT-reflective parameters (E-cadherin, vimentin, slug) to further elucidate the importance of resveratrol as well as its effect *via* β1-integrin receptors and the NF-κB signaling pathway.

**FIGURE 8:**
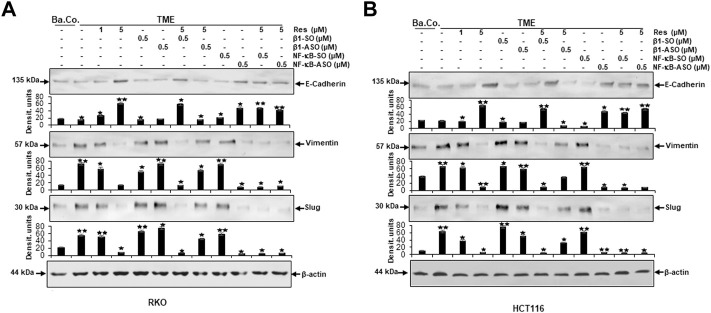
Impact of resveratrol or/and ASO against β1-integrin or against NF-κB on TME-promoted activation of EMT-linked biomarkers in CRC cells. 3D-alginate RKO **(A)** and HCT116 **(B)** CRC cells were detected against E-cadherin, vimentin, slug and loading controlled with β-actin after 10–14 days of treatment. *X*-axis: untreated (Ba.Co.), TME control, resveratrol (1, 5 µM), β1-SO/ASO (0.5 µM), NF-κB-SO/ASO (0.5 µM) or combination of 0.5 µM SO/ASO and resveratrol (5 µM). *Y*-axis: Densitometric units (*y*-axis) complementing Western blot results and for analysis, data were compared to TME control: *p < 0.05 and **p < 0.01 were considered statistically significant.

The expression of E-cadherin, representing epithelial phenotype, was low in untreated baseline control (Ba.Co.) and untreated TME control as well as in TME culture treated with β1-SO (0.5 µM) or β1-ASO (0.5 µM) alone (Figures 8A,B). However, resveratrol when added developed a concentration-dependent (1, 5 µM), strong enhancing effect of E-cadherin expression in both, RKO (Figure 8A) and HCT116 (Figure 8B) cells, compared to cells of the TME control. As this increase was also distinct with combined administration of resveratrol (5 µM) and β1-SO (0.5 µM) to the TME, β1-SO proved to be a reliable control reagent. When TME was treated with resveratrol (5 µM) and β1-ASO (0.5 µM), though, E-cadherin expression remained down-regulated because resveratrol could not exert its full epithelial-stabilizing effect in both CRC cell lines (Figures 8A,B). It was noted that treatment with NF-κB-SO (0.5 µM) did not alter the low E-cadherin level in TME. In contrast, addition of NF-κB-ASO (0.5 µM) to the TME, resulted in down-regulation of inflammatory spread and, concurrently, increased E-cadherin expression. Furthermore, resveratrol-treatment (0.5 µM) of NF-κB-SO/-ASO-TME (0.5 µM) also reduced inflammation and markedly promoted epithelial features of RKO or HCT116 cells (Figures 8A,B). The mesenchymal markers vimentin and slug showed opposite results. Both biomarkers were significantly increased in TME compared to baseline control (Ba.Co.) and not significantly affected by β1-SO/-ASO addition (0.5 µM each) in both CRC cell lines (Figures 8A,B). Impressively, resveratrol treatment led to a significant down-regulation of these parameters, which also persisted with combined treatment of resveratrol and β1-SO (0.5 µM). With β1-integrin knockdown by β1-ASO (0.5 µM), however, resveratrol (5 µM) could no longer fully exert its anti-EMT effects, what resulted in a strong increase of vimentin and slug (Figures 8A,B). Whereas the high expression of both biomarkers in TME was unaffected by treatment with NF-κB-SO (0.5 µM), both, NF-κB knockdown (0.5 µM NF-κB-ASO) and the combination treatment of resveratrol and NF-κB-SO or NF-κB-ASO lead to strong down-regulation in HCT116 and RKO, compared with TME control (Figures 8A,B). For all examinations, β-actin served as an internal control. In summary, resveratrol showed a considerable anti-EMT effect (Figures 8A,B) and, supported by the immunofluorescence results (Figures 1, 3), these findings suggest the use of β1-integrin receptors by resveratrol to exert its anti-invasion impact in CRC cells which was not cell line specific.

### Resveratrol targets β1-integrin receptors, modulates TME-induced NF-κB phosphorylation and NF-κB-associated migration, metastasis and apoptosis proteins in the same manner as NF-κB-ASO in CRC cells

**FIGURE 9:**
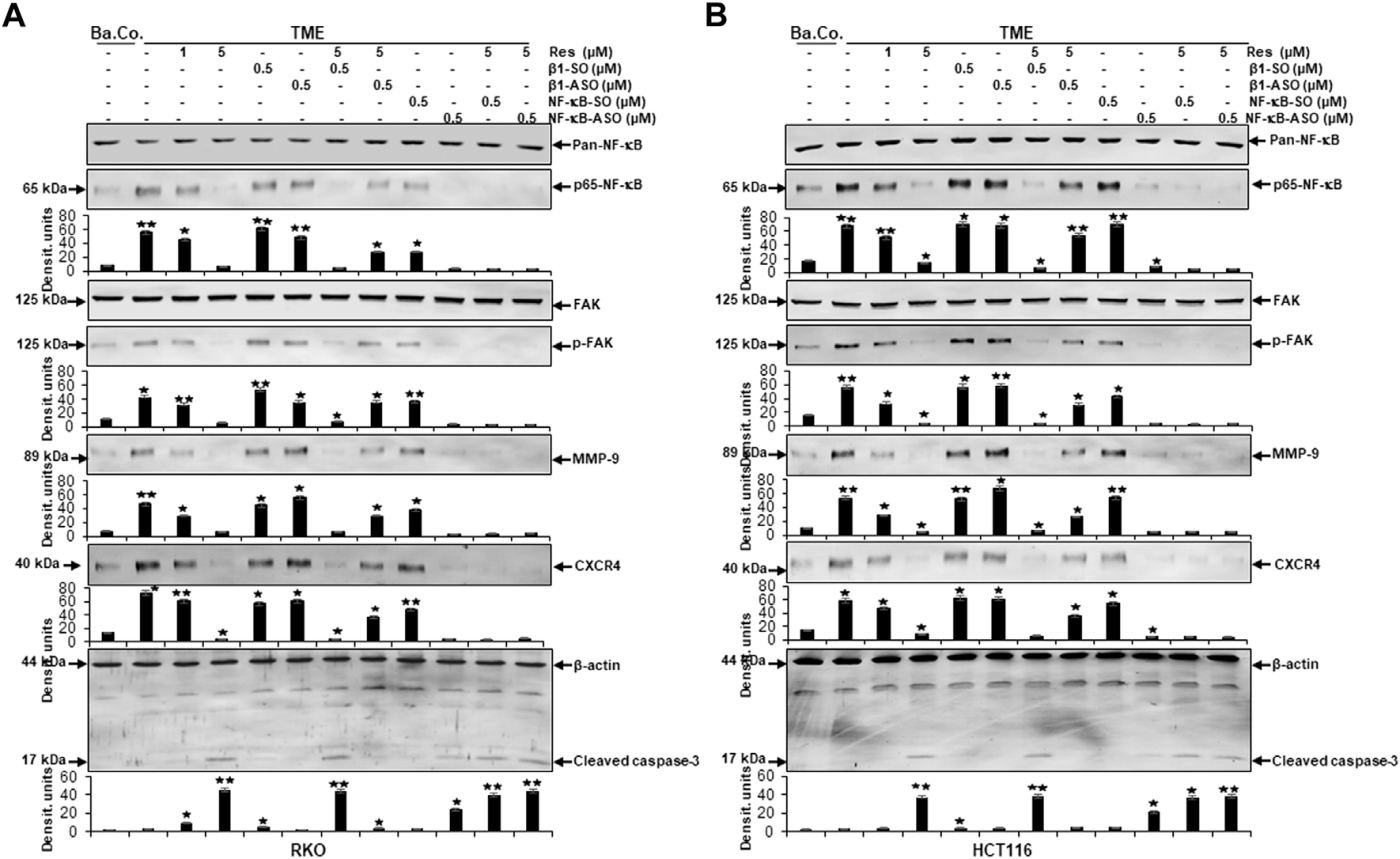
Impact of resveratrol or/and ASO against β1-integrin or against NF-κB on TME-promoted activation of metastasis- and apoptosis-linked biomarkers in CRC cells. Serum-starved RKO **(A)** and HCT116 **(B)** CRC cells, grown in 3D-alginate and differently treated for 10–14 days [untreated (Ba.Co.), TME control, resveratrol (1, 5 µM), β1-SO/ASO (0.5 µM), NF-κB-SO/ASO (0.5 µM) or combination of 0.5 µM SO/ASO and resveratrol (5 µM); *x*-axis] were investigated with antibodies against pan-NF-κB, p65-NF-κB, FAK, p-FAK, MMP-9, CXCR4 and cleaved caspase-3. β-actin served as loading control and statistical significance is shown by: *p < 0.05 and **p < 0.01 compared with the TME control. *Y*-axis: Densitometric units (*y*-axis) complementing Western blot results.

In the next step, same Western blot samples as used before were examined for the influence of resveratrol’s unfolding effects *via* β1-integrin receptors on metastasis and apoptosis markers. In addition, the effects of NF-κB knockdown (with NF-κB/p65-subunit-ASO as outlined in Material and Methods) were also taken into account, whereby RKO (Figure 9A) and HCT116 (Figure 9B) presented similar results:

Compared to untreated baseline control (Ba.Co.), TME significantly promoted the expression of phosphorylated NF-κB (p-NF-κB), phosphorylated FAK (p-FAK), MMP-9 as well as CXCR4 in both CRC cell lines, which are all known as inflammation- and metastasis-related factors. This effect was confirmed in both, the untreated TME and the control treatments with 0.5 µM β1-SO or 0.5 µM β1-ASO alone (Figures 9A,B). Resveratrol’s concentration-dependent (1, 5 µM) down-regulation of all these parameters was surprising and equally observed in the presence of the control substance β1-SO (0.5 µM). In β1-ASO (0.5 µM) treated CRC cells, resveratrol was unable to exert its effect properly though, so that the expression of metastasis-related and inflammatory biomarkers was up-regulated instead of suppressed. Furthermore, NF-κB-SO (0.5 µM) addition barely affected TME, whereas NF-κB knockdown with 0.5 µM NF-κB-ASO or combined treatment with resveratrol and NF-κB-SO or NF-κB-ASO suppressed indicators of inflammation and invasion in RKO and HCT116 (Figures 9A,B). In line with these observations, the complementary study of apoptosis presented inverse results with low caspase-3 detection in all control samples, meaning baseline control (Ba.Co.), TME control as well as TME-CRC cells treated with 0.5 µM β1-SO or 0.5 µM β1-ASO (Figures 9A,B). Also in this regard, resveratrol showed a major modulatory effect by significantly increasing caspase-3 levels in a concentration-dependent (1, 5 µM) manner, both in TME-CRC cells and in β1-SO-treated (0.5 µM) TME-CRC cells, which was inhibited by β1-integrin knockdown (0.5 µM β1-ASO), visible by very low caspase-3 expression. Although the apoptosis rate was low in TME control treated with 0.5 µM NF-κB-SO, caspase-3 expression increased by NF-κB knockdown (0.5 µM NF-κB-ASO) and was maintained at a high level by resveratrol (5 µM) in both, NF-κB-SO- and NF-κB-ASO-treated RKO and HCT116 cells (Figures 9A,B).

The results described were supported by the consistent expression of non-phosphorylated FAK and NF-κB (pan-NF-κB) in all treatments, serving as a reference control while the uniform β-actin expression functions as a loading control. Overall, these findings indicate the utilization of β1-integrin receptors by resveratrol to unfold its strong anti-metastasis effects in CRC cells. Furthermore, it is noticeable that resveratrol developed its strongly anti-apoptotic effect even beyond NF-κB knockdown, suggesting resveratrol to powerfully complement its action. In summary, all Western blot results presented (Figures 6–9) were reproducible in both cell lines, HCT116 and RKO.

## Discussion

After revealing the significance of β1-integrin in the context of resveratrol’s anti-viability and anti-proliferative impact on CRC cells *in vitro* in our latest work (Brockmueller et al., 2022), the present study was devoted to the role of β1-integrin in association with anti-invasive and anti-metastatic resveratrol treatment, focusing on metastasis formation to detect a potential association. The central new, and in two CRC cell lines reproducible, insights gained within our study were: 1) resveratrol uses β1-integrin receptors to shift the balance from mesenchymal to epithelial morphology in CRC cells; 2) resveratrol uses β1-integrin receptors to down-regulate invasion as well as metastasis of CRC cells; 3) furthermore, resveratrol uses β1-integrin receptors to suppress inflammation in CRC cells; 4) and finally, resveratrol synergistically amplifies NF-κB knockdown, thereby increasing the anti-inflammatory effect on CRC cells.

**FIGURE 10:**
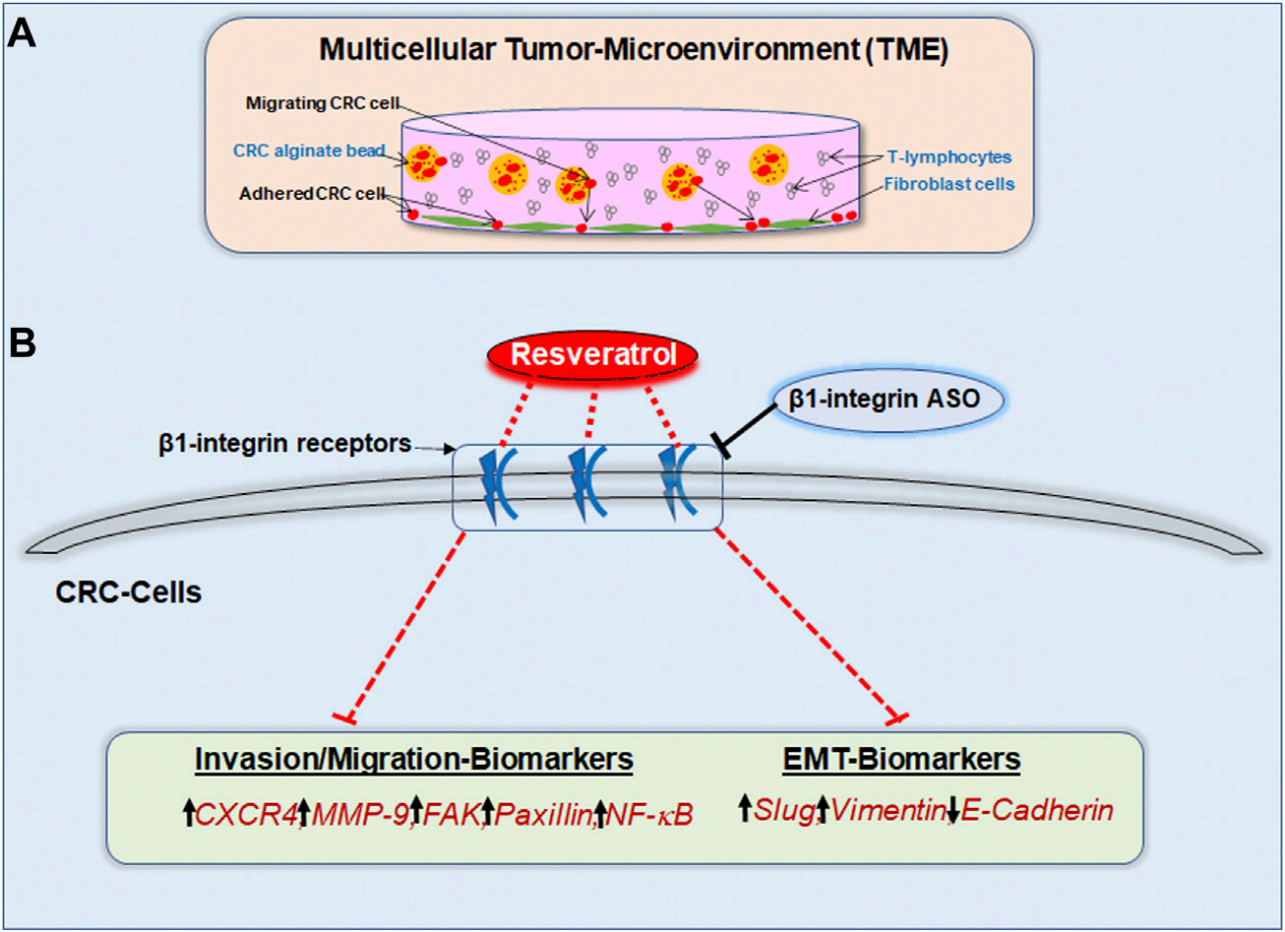
**(A)** Study model of the pro-inflammatory 3D-tumor microenvironment consisting of a fibroblast monolayer, floating T-lymphocytes in cell culture medium and CRC cells encapsulated in alginate. **(B)** Graphical outcome of resveratrol’s anti-invasive, anti-metastasis and anti-EMT effects by using β1-integrin receptors for signal transmission. The black arrows illustrate the biomarker situation within unaffected CRC cells. The red dashed lines show the modulatory effect of resveratrol on invasion-, migration- as well as EMT-parameters *via* β1-integrin receptors. β1-integrin-ASO can weaken this effect of resveratrol through β1-integrin receptor inhibition.

EMT together with its associated phenotype alteration pave the way for tumor cells to metastasize and invade peripheral tissue, serving as an indicator for severe disease progression. The EMT process, when tumor cells lose E-cadherin as epithelial organizer and instead take on a mesenchymal character, symbolized by slug overexpression, plays a strong role in CRC cells. It is known that EMT-promoting factors, such as cytokines and growth factors, are mainly generated by the cross-talk between tumor and immune cells (Buhrmann et al., 2020; Vuletić et al., 2021). Therefore, the ability of resveratrol to interrupt this exchange in our 3D-alginate model (Figure 10) represents a very important finding and simultaneously serves as encouragement for further investigation. The present results confirmed that resveratrol is able to prevent EMT-driven changes in the TME mainly by up-regulating the expression of E-cadherin and the down-regulation of the expression of multifunctional regulatory adhesion protein paxillin, intermediate filament vimentin and EMT-associated master transcription factor slug. These results confirm that paxillin as known downstream intracellular target protein of FAK, controls the interaction of integrin and extracellular ligands (Deakin et al., 2012). Interestingly, stimulation of paxillin has also been reported to alter the functional composition of focal adhesions, thereby significantly increasing cell motility (Devreotes and Horwitz, 2015). As these EMT-inhibitory properties are rendered impossible by β1-integrin knockdown, it can be assumed that resveratrol uses β1-integrin receptors in order to shift the balance from mesenchymal to epithelial phenotype in CRC cells, strongly highlighting the potential of resveratrol as an effective agent for cancer and metastasis prophylaxis *via* the utilization of β1-integrin receptors. The significance becomes even clearer in light of the fact that paxillin is considered a migration marker in CRC cells due to its direct connection to EMT (Wen et al., 2020).

Moreover, further evidence that previously described the relationship between integrin family members and EMT is provided by the finding of β4-integrin being responsible for the organization of vimentin filaments in lung cancer cells (Colburn and Jones, 2018). In addition, αvβ3-integrin is known to be a necessary essential condition for slug activation in breast cancer cells (Desgrosellier et al., 2014). To concretely link molecular events with the clinically diagnosable course of a cancer disease, ten factors of tumorigenesis, that have become known as hallmarks of cancer (Hanahan and Weinberg, 2011), were noted by Hanahan and Weinberg further highlighting the importance of inflammation and invasion. Recently, Welch and Hurst complemented these factors by adding four hallmarks of metastasis including motility/invasion, modulated microenvironment, plasticity and colonization (Welch and Hurst, 2019), underlining their high relevance in the search of alternative treatment strategies for targeting metastatic tumors. Invasion and metastasis involve cell adhesion, cell growth, and degradation of tissue barriers and are inseparable in tumor progress (Gupta et al., 2010). Our invasion assays confirmed the already demonstrated increasement of the invasion capacity of CRC cells in TME and highlighted how this process was down-regulated by resveratrol treatment. Impressively, the metastasis-inhibiting impact of resveratrol was significantly abrogated by β1-integrin knockdown, what was further reinforced by Western blot results. Moreover, resveratrol treatment alone was able to down-regulate the metastasis-related factors including FAK, of which cascade activation is known to be integrin-dependent (Lipfert et al., 1992; Cheng et al., 2021) as well as CXCR4, shown to up-regulate αvβ6-integrin in CRC cells, thus promoting metastasis (Wang et al., 2014). Furthermore, resveratrol was able to up-regulate apoptosis-marker caspase-3, but all these effects were not detectable in β1-integrin knockdown CRC cells.

Interestingly, the down-regulation of caspase-3 by integrin knockdown in *Helicobacter pylori* infected gastric epithelial cells has been previously reported too (Li et al., 2021). Overall, it is apparent that resveratrol reduced CRC cell invasion and migration *via* β1-integrin receptors, what leads to the assumption that its signaling pathway might be a suitable co-treatment for CRC that is urgently needed, since at least 50% of CRC cases are associated with metastases (Vatandoust et al., 2015). The aforementioned hallmarks of CRC progression, EMT and metastasis, are promoted by chronic inflammatory processes (Vuletić et al., 2021) indicating the great importance of NF-κB, which is considered a major inflammatory and tumorigenesis marker (Ko et al., 2017) in its phosphorylated, thereby activated form. Our results clearly demonstrated that resveratrol suppresses NF-κB activation in pro-inflammatory TME, what is consistent with previous results (Buhrmann et al., 2020). However, a new observation found is that both, immunofluorescence and Western blot analysis, reproducibly displayed an abolition of resveratrol’s anti-inflammatory effect by β1-integrin knockdown, making it obvious that resveratrol uses β1-integrin receptors to exert its inflammation-suppressing effect in CRC cells, in turn, leading to lower metastatic potential. On this background, the β1-integrin pathway represents a promising target in the fight against inflammation-based cancer.

Moreover, due to the strong association between phosphorylated NF-κB and cancer progression, we finally investigated on resveratrol’s impact on CRC cells in an inflammatory environment when NF-κB was knocked down. Here, we found that NF-κB knockdown led to a decreased inflammatory spread and invasion capacity in both CRC cell lines HCT116 and RKO, whereby resveratrol treatment of NF-κB down-regulated CRC cells significantly enhanced their ability to act in a synergistic, powerful anti-inflammatory way. Based on this finding, it is worth considering the potential of resveratrol in the future with regard to the treatment of cancer and other chronic inflammatory diseases such as rheumatoid arthritis, where NF-κB activation also plays a crucial role or Crohn’s disease, where dysregulation of NF-κB, physiologically necessary for intestinal homeostasis, triggers an inflammatory cascade (Nissim-Eliraz et al., 2021; Mueller et al., 2022). The fact that integrin receptors physiologically occur and are necessary in the embryonic development is also exploited by malignant cancer cells in the event of disease, thus for treatment of hepatocellular carcinoma, reduction of α2-, β1-, and β3-integrin expression has been proposed as a possible option (Relja et al., 2011). Also in neuroblastoma cells, up-regulated α2-, α3- and β1-integrin expression was shown and invasion and migration could be inhibited mainly by silencing β1-integrin (Lee et al., 2013). Moreover, the correlation between β1-integrin upregulation and the migration of triple negative breast cancer cells has been demonstrated in previous research (Schlienger et al., 2015).

Altogether, β1-integrin has already been shown to act as a fundamentally important receptor in various types of cancer in the digestive tract and other organ systems. Extremely high CRC case numbers underline the urgency to explore the exact mechanisms underlying colorectal cancer, whereby already existing studies of other cancer types support the potential of our observations made in HCT116 and RKO cells. Our findings show evidence that high β1-integrin expression in cancer cells can be considered as a promising target to be used in CRC therapy. For the first time we suggest to rather use β1-integrin receptors as promising gateways for CRC co-treatment with the bio-active phytopharmaceutical resveratrol, especially due to its anti-invasive potential.

## Conclusion

The presented results shed light on β1-integrin’s role in resveratrol-mediated increased E-cadherin, caspase-3 expression and decreased phosphorylated NF-κB, phosphorylated FAK, vimentin, slug, paxillin, CXCR4, MMP-9 expression (Figure 10) in HCT116 and RKO cells leading to a noticeable reduction in their invasion and metastasis activity. Resveratrol has been shown to stabilize epithelial balance and to prevent metastasis of CRC cells, whereby these effects were significantly weakened by the knockdown of β1-integrin. Overall, we conclude that resveratrol exerts its anti-inflammatory, anti-invasive and thus cancer-inhibiting effect, to a relevant extent, *via* β1-integrin receptors. Therefore, we are convinced of the helpfulness of clinical studies to determine whether β1-integrin is suitable as a tumor marker for CRC and to emphasize the great opportunity of innovative, resveratrol-based drugs against highly metastatic CRC *via* the β1-integrin action mechanism.

## Data Availability

The original contributions presented in the study are included in the article/Supplementary Material, further inquiries can be directed to the corresponding author.
